# P2Y6 Receptor-Mediated Microglial Phagocytosis in Radiation-Induced Brain Injury

**DOI:** 10.1007/s12035-015-9282-3

**Published:** 2015-06-23

**Authors:** Yongteng Xu, Weihan Hu, Yimin Liu, Pengfei Xu, Zichen Li, Rong Wu, Xiaolei Shi, Yamei Tang

**Affiliations:** 1 Department of Neurology, Sun Yat-Sen Memorial Hospital, Sun Yat-Sen University, Number 107, Yan Jiang Xi Road, Guangzhou, Guangdong Province 510120 China; 2 Key Laboratory of Malignant Tumor Gene Regulation and Target Therapy of Guangdong Higher Education Institutes, Sun Yat-Sen University, Guangzhou, 510120 China; 3 Department of Radiation Oncology, Cancer Center of Sun Yat-Sen University, Guangzhou, 510120 China; 4 Department of Radiation Oncology, Sun Yat-Sen Memorial Hospital, Sun Yat-Sen University, Guangzhou, 510120 China

**Keywords:** P2Y6 receptor, Microglial phagocytosis, Radiation-induced brain injury, Myelin, Neuronal apoptosis, Rac1, MLCK

## Abstract

Microglia are the resident immune cells and the professional phagocytic cells of the CNS, showing a multitude of cellular responses after activation. However, how microglial phagocytosis changes and whether it is involved in radiation-induced brain injury remain unknown. In the current study, we found that microglia were activated and microglial phagocytosis was increased by radiation exposure both in cultured microglia in vitro and in mice in vivo. Radiation increased the protein expression of the purinergic receptor P2Y6 receptor (P2Y6R) located on microglia. The selective P2Y6 receptor antagonist MRS2578 suppressed microglial phagocytosis after radiation exposure. Inhibition of microglial phagocytosis increased inhibitory factor Nogo-A and exacerbated radiation-induced neuronal apoptosis and demyelination. We also found that the levels of protein expression for phosphorylated Ras-related C3 botulinum toxin substrate 1 (Rac1) and myosin light chain kinase (MLCK) were elevated, indicating that radiation exposure activated Rac1 and MLCK. The Rac1 inhibitor NSC23766 suppressed expression of MLCK, indicating that the Rac1-MLCK pathway was involved in microglial phagocytosis. Taken together, these findings suggest that the P2Y6 receptor plays a critical role in mediating microglial phagocytosis in radiation-induced brain injury, which might be a potential strategy for therapeutic intervention to alleviate radiation-induced brain injury.

## Introduction

Primary tumors affecting the head, neck, and brain as well as brain metastases occurring in 20–40 % of patients with cancer account for significant morbidity and mortality [1, 2]. Radiation therapy is one of the most effective treatment modalities for these types of tumors [3, 4]. Radiotherapy, although helpful in the management of central nervous system (CNS) and head and neck tumors, can cause devastating injury to normal CNS tissues [5, 6]. Currently, few effective strategies alleviate this radiation-induced brain injury. Although the exact pathogenic mechanisms of radiation-induced brain injury remain largely unknown, studies have demonstrated that microglia may play a pivotal role by releasing proinflammatory factors that induce an inflammatory response when activated by radiation [7, 8]. In addition to this inflammatory response aspect, microglia are also professional phagocytes in the CNS, maintaining homeostasis of the nervous system [9]. Following a CNS injury, microglia become activated and phagocytose dying cells, myelin debris, and apoptotic cells [10–12]. The efficient clearance of apoptotic cells may prevent the accumulation of necrotic cells and the subsequent release of detrimental intracellular components from these cells. Removal of myelin debris also helps to clear inhibitory factors, such as neurite outgrowth inhibitor (Nogo)-A, which block differentiation of oligodendrocyte precursor cells to oligodendrocytes, impairing remyelination [13]. In addition to this beneficial effect of microglial phagocytosis, evidence indicates that activated microglia mediate phagocytosis of viable neurons, leading to neuronal loss and death in models of focal brain ischemia and AIDS dementia [14, 15]. Activated and phagocytic microglia are also observed accumulated at the lesion/spinal cord interface in an irradiated hemisectioned spinal cord [12]. However, the mechanisms underpinning microglial phagocytosis in the pathogenesis of radiation-induced brain injury remain to be elucidated. Some studies have shown that extracellular uridine diphosphate (UDP) released by injured cells serves as an “eat me” signal and acts on P2Y6 receptors to initiate microglial phagocytosis [16]. Whether mediating UDP/P2Y6 signaling and microglial phagocytosis can alleviate radiation-induced brain injury requires further investigation.

In the current study, we demonstrated that microglial phagocytosis was activated after radiation both in vitro and in vivo. Radiation increased the expression of P2Y6 receptors, and this increase was inhibited by a P2Y6 receptor-specific antagonist. Radiation caused neuronal apoptosis and induced a remyelination deficiency, both of which were aggravated by inhibiting UDP/P2Y6R signaling and microglial phagocytosis. We also determined that the Ras-related C3 botulinum toxin substrate 1 (Rac1)-myosin light chain kinase (MLCK) pathway was involved in P2Y6R-mediated microglial phagocytosis in radiation-induced brain injury.

## Materials and Methods

### Ethical Statement

All experiments were approved by the ethical committee of Sun Yat-Sen University. All procedures followed the guidelines recommended by the Chinese National Institute of Health for humane care, which complies with the National Institutes of Health’s *Guide for the Care and Use of Laboratory Animals*. All efforts were made to minimize animal suffering, to reduce the number of animals used, and to utilize alternatives to in vivo techniques.

### Animals and Cell Culture

Adult male BALB/c mice (weighing 18–22 g) were obtained from the Laboratory Animal Center of Sun Yat-Sen University. Primary microglia were cultured according to a previously described method [17]. Briefly, a mixed glial culture was prepared from neonatal BALB/c mice and maintained for 10–20 days in medium (DMEM; Life Technologies, CA, USA) containing 10 % fetal calf serum, 10 U/mL penicillin, and 10 mg/mL streptomycin (Life Technologies). Microglia were obtained when cells floated over the mixed glial culture. The floating cells were collected by gentle shaking and transferred into appropriate dishes or glasses. The cells were used for Western blot, immunofluorescence, and phagocytosis assays.

### Irradiation Models

#### Cell Irradiation

The regimen of cell irradiation was based on our previous study. We tested the radiation dose effect of 3, 5, 8, and 10 Gy and found that inflammatory mediators did not change at 3 or 5 Gy, but increased significantly at 8 and 10 Gy. Because 8 Gy was previously shown to guarantee the cell survival, a single radiation dose of 8 Gy was used in the in vitro experiments. Primary microglia were cultured 24–36 h. The medium was replaced with serum-free medium 4 h before radiation exposure. The selective P2Y6 receptor antagonist MRS2578 (1 μmol/L; Sigma-Aldrich) was added 30 min before radiation. Cells were radiated with a linear accelerator using β ionizing radiation (Siemens, Germany).

#### Animal Irradiation

Adult male BALB/c mice were anesthetized with 10 % chloral hydrate (200 mg/kg, intraperitoneal injection). The mice were irradiated using a 6 MV linear accelerator with β ionizing radiation (Siemens, Germany) as described previously [17]. The head of the mouse, including the whole brain from the post-canthus line to the post-aurem line, was placed in the treatment field (2 × 2 cm^2^). Nonirradiated tissues were protected with a 1-cm-thick lead plate. In a previous study, a single dose of radiation higher than 25 Gy was applied to induce brain injury in rats [18]. Another study showed that microglial-derived COX-2 did not increase at doses less than 25 Gy [19]. Therefore, a single dose of 30 Gy was given at a rate of 3 Gy/min to the whole brain of mice in the present study. The animals were either sacrificed 7, 14, and 30 days after radiation exposure. These times were selected based on the results of our previous study showing that alterations in gene and protein expression occur as early as 3 days, morphological changes at 7 days. [17].

#### Antagonist Administration

Mice were anesthetized and positioned in a stereotaxic frame (Lanxing, China). A guide cannula was placed in the hippocampal CA3 region (coordinates calculated from bregma: anterior, −1.94 mm; lateral, 2.0 mm; ventral, 2.0 mm) and fixed with dental cement. One day after the operation, an injection cannula (1 mm longer than the guide cannula; Ruiwode, China) was inserted through the guide cannula. The P2Y6 receptor antagonist MRS2578 (100 μM/1 μL) was injected via the guide cannula 30 min before radiation and daily for the ensuing 6 consecutive days. Following the last injection of MRS2578, 1 μL of fluorescent microspheres (yellow green, 1 μm, Sigma-Aldrich) was injected. Control mice received the same volume of saline instead of MRS2578. The mice were sacrificed and perfused with 4 % paraformaldehyde 48 h after the microsphere injection.

#### Immunofluorescence

For immunocytochemistry, microglia were incubated with serum-free medium for 1 h before radiation exposure. For detection of florescent beads, primary microglia were incubated with fluorescent latex beads (1 μm, 0.0025 %, Sigma-Aldrich) in serum-free medium for 30 min at 37 °C with and without MRS2578 (100 μM). After incubation, the medium was removed, and cells were fixed with paraformaldehyde for 30 min. Cells were incubated with 0.5-mL 0.3 % Triton X-100 for 30 min after being washed three times. Cells were incubated at 4 °C overnight with rhodamine-phalloidin (1:40; Life Biotechnology). Fluorescence images were obtained with a fluorescence microscope (Olympus Optical, Japan).

For immunohistochemistry, the brains were removed, placed in 4 % paraformaldehyde for 12 h, transferred to a 30 % sucrose solution for 24 h, and then sliced for immunohistochemical analysis. For quantitative analysis of in vivo phagocytosis, sections (30 μm) containing microspheres were thoroughly washed (using a shaking plate, five times for 5 min each) with PBS to remove nonspecifically bound microspheres. Then, sections were incubated in 0.3 % Triton X-100 containing PBS (using a shaking plate, 10 times for 10 min each) and labeled with rabbit anti-Iba1 (1:500; Wako Pure Chemical Industries, Ltd.). Images were obtained with a confocal laser microscope (LSM5 Pascal, Carl Zeiss). The captured images were reconstructed and stacked in the Z dimension. The number of microspheres in the section was counted as an index of in vivo phagocytosis. Three sections containing the microspheres in each animal were analyzed, and at least three animals in each group were used. For double immunofluorescence staining, coronal brain slices (10 μm) were blocked with 5 % goat serum and incubated with rabbit polyclonal anti-P2Y6 receptor (1:50; Alomone), rabbit polyclonal anti-Iba1 receptor (1:500, Wako Pure Chemical Industries, Ltd.), mouse monoclonal anti-GFAP (1:500, Millipore), and mouse monoclonal anti-NeuN (1:500, Millipore) for 24 h at 4 °C. The appropriate secondary antibodies, including goat anti-rabbit Alexa 594, goat anti-mouse Alexa 594, and donkey anti-rabbit Alexa 488 (all purchased from Jackson ImmunoResearch), were incubated with the slices for 100 min at room temperature. After nuclear staining with 4′,6-diamidino-2-phenylindole (DAPI; Sigma-Aldrich) for 8 min, sections were coverslipped with glycerol, and fluorescent images were obtained with a fluorescence microscope (Olympus Optical, Japan).

### Fluorescence-Activated Cell Sorting

Phagocytosis was analyzed in vitro as previously described [20]. Briefly, microglia were plated at a density of 5 × 10^5^/dish. The fluorospheres (1-μm diameter green-yellow fluorescent microspheres, Sigma-Aldrich, prepared as described above) at 0.0025 % combined with the P2Y6R inhibitor or Rac1 inhibitor at various concentrations were added to the culture for 30 min at 37 °C. The cultures were washed with PBS at least three times. The cells were removed using EDTA solution and centrifuged at 500×*g* for 5 min. The cells were resuspended in 1-mL fluorescence activated cell sorting (FACS) buffer. The cell suspension was evaluated using a BD FACS Universal Loader (BD Biosciences) to analyze fluorescent intensity, which was detected using the FITC channel (488 nm). Data were analyzed with BD FACSuite software and expressed as the percentage of phagocytic cells. Alternatively, the cell suspension was separated using a FACSAria cell-sorting system (BD Biosciences) to determine the number of fluorospheres engulfed by phagocytic cells.

### Terminal Deoxynucleotidyl Transferase Dutp Nick end Labeling (TUNEL)

Apoptotic neuronal cells were detected via double staining with NeuN (1:250, Millipore) and TUNEL (FragEL DNA Fragmentation Detection Kit, Fluorescent-TdT Enzyme; Merck). In a separate experiment, mice were pretreated with MRS2578 (100 μM) or saline before radiation exposure and for the ensuing 7 days. The mice were then sacrificed and their brains sliced into coronal sections 10 μm thick. The ratio of apoptotic to live neuronal cells was calculated.

### Real-Time PCR

Total RNA isolation and real-time PCR were performed as previously described [17]. Total RNA was isolated from cells using the RNeasy Plus Mini Kit (TaKaRa), according to the manufacturer’s instructions. After reverse transcription, quantitative real-time PCR was performed using primers specific for the genes encoding TNF-α, IL-1β, IL-6, iNOS, and GAPDH. The following primers were used: iNOS, sense, 5′-CTC ACT GTG GCT GTG GTC ACC TA-3′, anti-sense, 5′-GGG TCT TCG GGC TTC AGG TTA-3′; IL-6, sense, 5′-GTC ACA GAA GGA GTG GCT AAG GA-3′, anti-sense, 5′-TAA CGC ACT AGG TTT GCC GAG TAG-3′; TNF-α, sense, 5′-TTG ACC TCA GCG CTG AGT TG-3′, anti-sense, 5′-CCT GTA GCC CAC GTC GTA GC-3′; IL-1β, sense, 5′-GCT GTG GCA GCT ACC TAT GTC TTG-3′, anti-sense, 5′-AGG TCG TCA TCA TCC CAC GAG-3′; GAPDH, sense, 5′-ATC TTC TTG TGC AGT GCC AG-3′, anti-sense, 5′-CGT TGA TGG CAA CAA TCT CC-3′. PCR was performed for 10 min at 95 °C, followed by 50 cycles for 10 s at 95 °C, annealing for 10 s at 60 °C, and extension for 20 s at 72 °C. The mRNA expression levels were assessed using a Roche LightCycler with a LightCycler FastStart DNA Master SYBR Green I kit. The results are expressed as the relative mRNA expression of the threshold cycle value and were normalized by parallel amplification of the endogenous control GAPDH. The target mRNA expression level in the control group (target mRNA/GAPDH value) was set to 100 %, and levels in other groups were converted to fold changes by comparing them to the control group.

### Western Blotting

Total protein was prepared and quantified using primary microglia or brain tissue from mice. The P2Y6 receptor expression level after radiation exposure and the radiation-induced p-Rac1, Rac1, and MLCK expression levels in microglia were evaluated using Western blot analysis. Briefly, 50 μg of total protein loaded in each lane was separated with sodium dodecyl sulfate polyacrylamide gel electrophoresis and transferred to a polyvinylidene fluoride membrane (Millipore, USA) using an electrophoretic transfer apparatus (Bio-Rad). The membrane was blocked with 5 % bovine serum albumin in Tris-buffered saline containing 0.05 % Tween 20 (TBST). The membrane was incubated with rabbit anti-mouse P2Y6 receptor polyclonal antibody (Alomone, Israel), rabbit anti-mouse Rac1 monoclonal antibody (Millipore, USA), rabbit anti-rabbit phosphorylation Rac polyclonal antibody (CST, USA), and rabbit anti-rabbit MLCK polyclonal antibody (Abgent, USA) in blocking solution overnight at 4 °C. After thorough washing in TBST, the membranes were incubated with secondary antibodies, goat anti-rabbit or goat anti-mouse IgG–horseradish peroxidase conjugate (LiankeBio, China), and the proteins were developed using the Enhanced Chemiluminescence detection system (Millipore, USA) with exposure to X-ray film. The relative densities of bands were analyzed with a gel imaging analysis system (Genetics Inc., USA).

### Electron Microscopy

Mice were anesthetized with 10 % chloral hydrate and perfused with paraformaldehyde (4 %). The hippocampus was removed and cut into small pieces (approximately 1 cubic millimeter). After primary fixation of brain sections in glutaraldehyde (2.5 %) prepared in phosphate buffer (0.1 M, pH 7.4) and paraformaldehyde (2 %) for 4 h, postfixation was conducted in osmium tetroxide (1 %) for 1.5 h, followed by dehydration and embedding in resin, DDSA, NMA, and DMP-30 medium overnight. The tissues were then baked at 65 °C for 48 h. Finally, the tissues were cut into thin sections (60–80 nm) using an ultramicrotome (Leica, UC6/FC6). The thin sections on copper mesh grids were stained with uranyl acetate and lead citrate for contrast. The brain sections were scanned using a transmission electron microscope (FEI, Tecnai G2 Spirit Twin).

### Statistical Analysis

All experiments were repeated at least three times, and the results are presented as the mean ± SEM. Statistical analyses were performed using a one-way ANOVA followed by Bonferroni corrections for multiple group comparisons or Student’s *t* test for comparisons between two groups with SPSS 16.0 software. Resulting *P* values <0.05 were considered statistically significant.

## Results

### Radiation Activates Microglia and Increases Phagocytosis

**Fig. 1:**
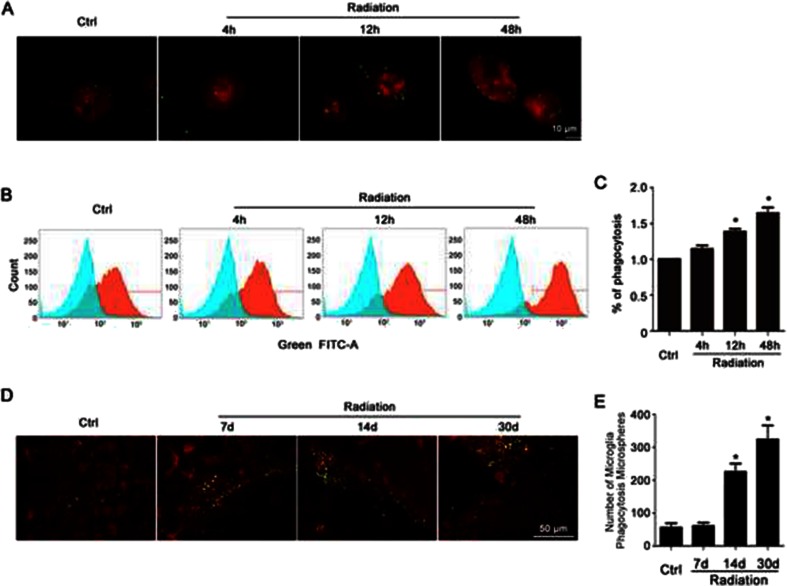
Radiation activates microglia and increases phagocytosis. **a** Cultured microglia preincubated with fluorescent latex beads (*green*) were irradiated, and then immunostained with Iba1 (*red*). **b** Cells treated as in **a** were subjected to flow cytometry. Representative images of the intensity of FITC-A staining (*orange* histograms) and control with no fluorescence (*green* histograms) were shown. **c** Quantification of percentage of microglia phagocytosing fluorescent latex beads was shown as mean ± SEM; *n* = 3; *P < 0.05 compared with unirradiated control group. **d** Representative images of fluorescent microspheres (*green*) attached or taken up by microglia (*red*, anti-Iba1) in the control and irradiated hippocampal CA3 regions. *Scale bar* 50 mm. **e** Quantitative analysis of phagocytosis in vivo was shown as mean ± SEM; *n* = 3; * *P* < 0.05 compared with unirradiated control group

To confirm that radiation activated microglia and to evaluate how microglial phagocytosis changed following radiation, cultured primary microglia were exposed to 8 Gy of β radiation. Latex beads (green in Fig. 1) were added to quantitatively evaluate microglial phagocytosis. The morphological alteration of microglia (Iba1, red in Fig. 1) was examined using immunofluorescence labeling. As shown in Fig. 1a, β radiation altered the morphology of microglia from ramified to amoeboid, with larger soma and thicker but shorter protrusions. Irradiated cells phagocytosed more beads than control cells did, showing a time-dependent increase. Microglial phagocytosis was further quantified with a phagocytosis assay using FACS. As shown in Fig. 1b, compared with the control group, there was a significant increase in microglial phagocytosis after radiation exposure. This increase was time dependent (Fig. 1c). Because the latex beads were added 30 min before microglia were fixed for the morphology examination or phagocytosis assay, the time the latex beads were incubated in all groups was identical. Therefore, phagocytosis, and not incubation time, accounted for the difference between the groups.

We also determined whether radiation facilitated microglial phagocytosis in vivo. BALB/c mice were irradiated with single dose of 30 Gy to the head. Fluorescent microspheres were injected into the hippocampus CA3 region 3 days before the animals were sacrificed for examination. The number of microspheres phagocytosed or attached to microglia was counted. As shown in Fig. 1d, e, the number of microspheres phagocytosed or attached to microglia significantly increased after radiation in a time-dependent manner. Taken together, these findings suggest that radiation markedly activated microglia and facilitated microglial phagocytosis both in vitro and in vivo.

### Radiation Upregulates P2Y6 Receptor Expression

**Fig. 2:**
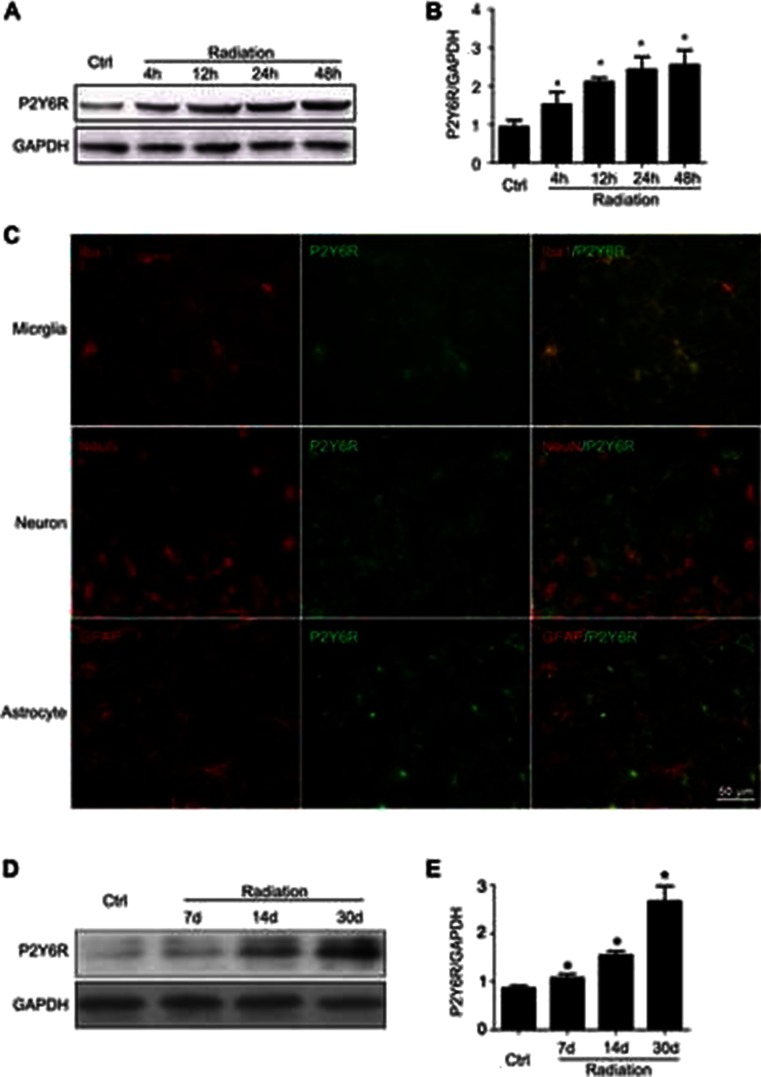
Radiation upregulates P2Y6 receptor expression***.*** **a** P2Y6R expression in primary mouse microglia was determined by Western blotting 4 h, 12 h, 1, and 2 days after radiation. **b** Densitometric analysis of P2Y6R versus GAPDH was shown as mean ± SEM; *n* = 3; * *P* < 0.05 compared with unirradiated control group. **c** P2Y6R colocalize with Iba1^+^ microglia, but not in NeuN^+^ neuron, or GFAP^+^ astrocytes in BALB/c mice that received a single dose of 30 Gy radiation*. Scale bar* 50 μm. **d** P2Y6R expression in mice that received a single 30 Gy radiation dose. **e** Densitometric analysis of P2Y6R versus GAPDH. Mean ± SEM; *n* = 3; * *P* < 0.05 compared with unirradiated control group

After demonstrating that radiation induced microglia activation and increased phagocytosis, we next explored the underlying mechanisms. The purinoceptor P2Y6, a member of P2 receptor family, reportedly contributes mainly to microglial phagocytosis in the CNS [20]. Therefore, we examined the expression and function of the P2Y6 receptor in irradiated microglia in vitro and in vivo. As shown in Fig. 2a, b, the level of P2Y6R protein expression in freshly isolated microglia was significantly increased after single dose of radiation (8 Gy). This increase began 4 h after radiation and remained elevated for 2 days. In vivo experiments confirmed the in vitro data. Whole brain irradiation of BALB/c mice at a single dose of 30 Gy. We examined the cell types in which the P2Y6R protein expressed in response to radiation. Immunolabeling of the irradiated brain revealed that the P2Y6R expression increase wasconfined to microglia and was not detected in neurons or astrocytes (Fig. 2c). Irradiation increased P2Y6R protein expression, beginning 7 days and remaining for 30 days after radiation exposure (Fig. 2d, e). Thus, P2Y6R protein expression levels are upregulated after radiation both in vitro and in vivo*.*

**Fig. 3:**
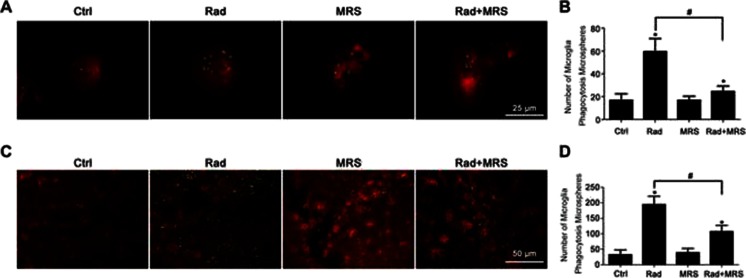
P2Y6 receptor mediates radiation induced microglial phagocytosis. **a** Cultured microglia incubated with fluorescent latex beads were irradiated, or pretreated with specific P2Y6R antagonist MRS2578 before radiation. Cells were immunostained with Iba1. **b** Quantitative analysis of phagocytosis in vitro. Data were shown as mean ± SEM; *n* = 3; * *P* < 0.05, compared with control group; # *P* < 0.05 compared with radiation group. **c** Representative images of fluorescent microspheres (*green*) attached or taken up by microglia (*red*, anti-Iba1) at hippocampal CA3 regions in control, irradiated, MRS2578 treated mice or irradiated mice treated with MRS2578. *Scale bar* 50 μm. **d** Quantitative analysis of phagocytosis in vivo was shown as mean ± SEM; *n* = 3; *P < 0.05 compared with control group. # *P* < 0.05 compared with radiation group

### Inhibition of P2Y6 Receptor Antagonizes Radiation-Induced Phagocytosis

Thus far, we demonstrated that radiation induced an upregulation in P2Y6R protein expression and a parallel increase in microglial phagocytosis both in vitro and in vivo. We next examined whether the radiation-induced enhancement of microglial phagocytosis was mediated by the P2Y6R. As shown in Fig. 3a, b, the radiation-induced increase in microglial phagocytosis was blocked when microglia were preincubated with the selective P2Y6R antagonist MRS2578 (1 μM) 1 h before radiation. However, MRS2578 alone did not significantly alter phagocytosis in control microglia receiving no radiation. The effect of MRS2578 was also examined in irradiated mice. MRS2578 (5 μmol/kg, once daily) was injected into the hippocampus CA3 region for 7 consecutive days, starting on the day of radiation exposure. Fluorescent microspheres were injected 3 days before the mice were sacrificed. As shown in Fig. 3c, d, MRS2578 significantly inhibited the radiation-induced increase in microglial phagocytosis in these mice. These results indicate that the P2Y6R is involved in the radiation-induced phagocytosis by microglia. Thus, pharmacological inhibition of the P2Y6 receptor may significantly suppress the radiation-induced increase in microglial phagocytosis.

### P2Y6 Receptor Antagonism Inhibits Microglial Phagocytosis and Exacerbates Brain Injury in Irradiated Mice

**Fig. 4:**
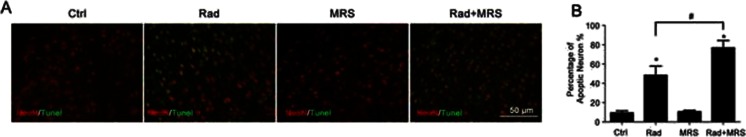
Inhibition of P2Y6 receptor exacerbates neuronal apoptosis. **a** Representative cortex sections were immunostained with anti-NeuN (*red*) and TUNEL (*green*). *Scale bar* 50 μm. **b** Quantitative analysis of percentage of apoptotic neurons were shown as mean ± SEM; *n* = 3; * *P* < 0.05 compared with control group; # *P* < 0.05 compared with radiation group

**Fig. 5:**
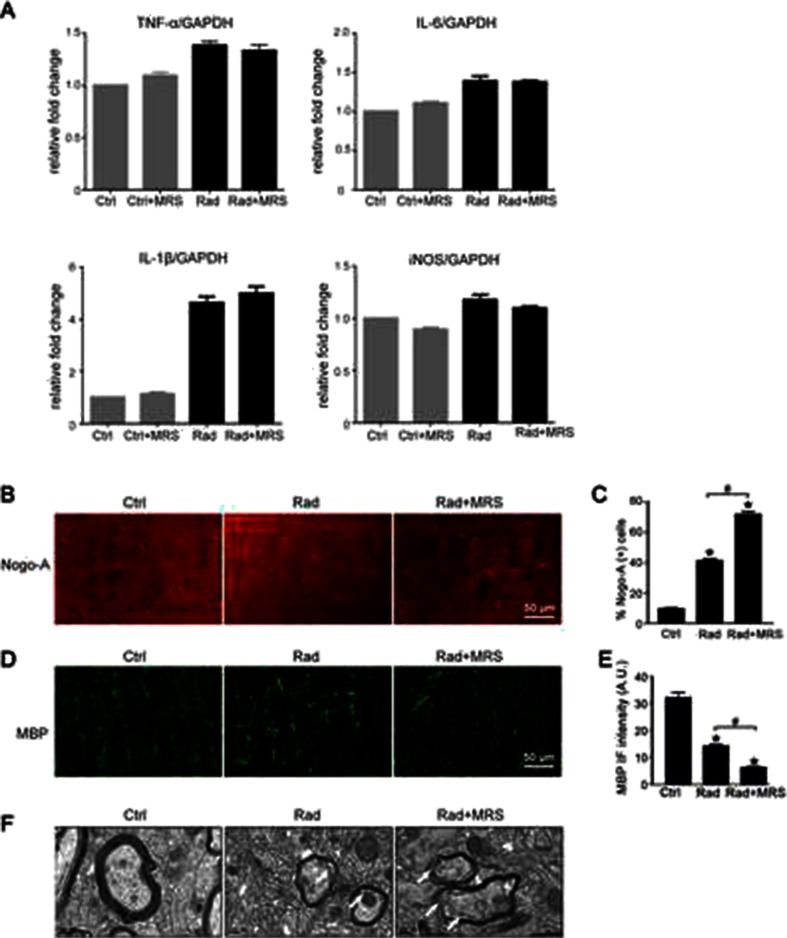
P2Y6 receptor antagonism exacerbates radiation induced brain injury. **a** Lysates of microglia treated as indicated for 24 h (ctrl, control; rad, radiation; MRS, MRS2578) were subjected to real-time quantitative PCR with the primers of TNF-α, IL-6, IL-1β, and iNOS (GAPDH as loading control). The results were quantified and the levels in control group compared to GAPDH were set 100 %, the fold changes in other groups were shown. **b** Representative cortex sections were immunostained with anti-Nogo-A antibody (*red*). *Scale bar* 50 μm. Quantified data were shown in **c** as mean ± SEM; *n* = 3; * *P* < 0.05 compared with control group; # *P* < 0.05 compared with radiation group. **d** Representative cortex sections were immunostained with anti-MBP (*green*) antibody. *Scale bar* 50 μm. Statistical data were shown in **e** as mean ± SEM; *n* = 3; * *P* < 0.05 compared with control group; # *P* < 0.05 compared with radiation group. **f** Representative images of demyelination were shown by electron microscope scanning after radiation. *Arrows* indicated the demyelination changes after radiation or pretreated with MRS2578

Having demonstrated that microglial phagocytosis increased after radiation and that the P2Y6R modulated this process, we next explored the function of this microglial phagocytosis, that is, we investigated whether the radiation-induced increase in microglial phagocytosis was beneficial or detrimental. Mice irradiated with a single dose of 30 Gy to the head showed increased apoptotic neurons in the cortex (Fig. 4), as identified by increased TUNEL staining colocalized with NeuN 14 days after radiation exposure. Administration of the P2Y6R antagonist MRS2578 significantly increased the number of apoptotic neurons. In a previous study, we showed that microglia are activated by radiation and release proinflammatory factors, which contribute to brain injury [17]. Because MRS2578 did not influence the release of inflammatory mediators, such as TNF-α, IL-1β, IL-6, or iNOS (Fig. 5a), the exacerbation of neuronal injury observed in the present study was attributed mainly to the decrease in microglial phagocytosis. In addition, the radiation-induced inhibition of microglial phagocytosis increased accumulation of Nogo-A (Fig. 5b, c) and exacerbated demyelination as shown by MBP staining (Fig. 5d, e). Our findings from the electron microscopy analysis confirmed that demyelination occurred after radiation and was aggravated by the inhibition of microglial phagocytosis induced by blocking the P2Y6 receptor with MRS2578 (Fig. 5f). Taken together, these data suggest that microglial phagocytosis contributes to the removal of apoptotic neurons and the inhibitory factor Nogo-A. The efficient clearance of the injury-induced debris is critical for the recovery of the irradiated brain that occurs through facilitating remyelination.

### Rac1-MLCK Pathway Is Involved in P2Y6 Receptor-Mediated Microglial Phagocytosis After Radiation Exposure

**Fig. 6:**
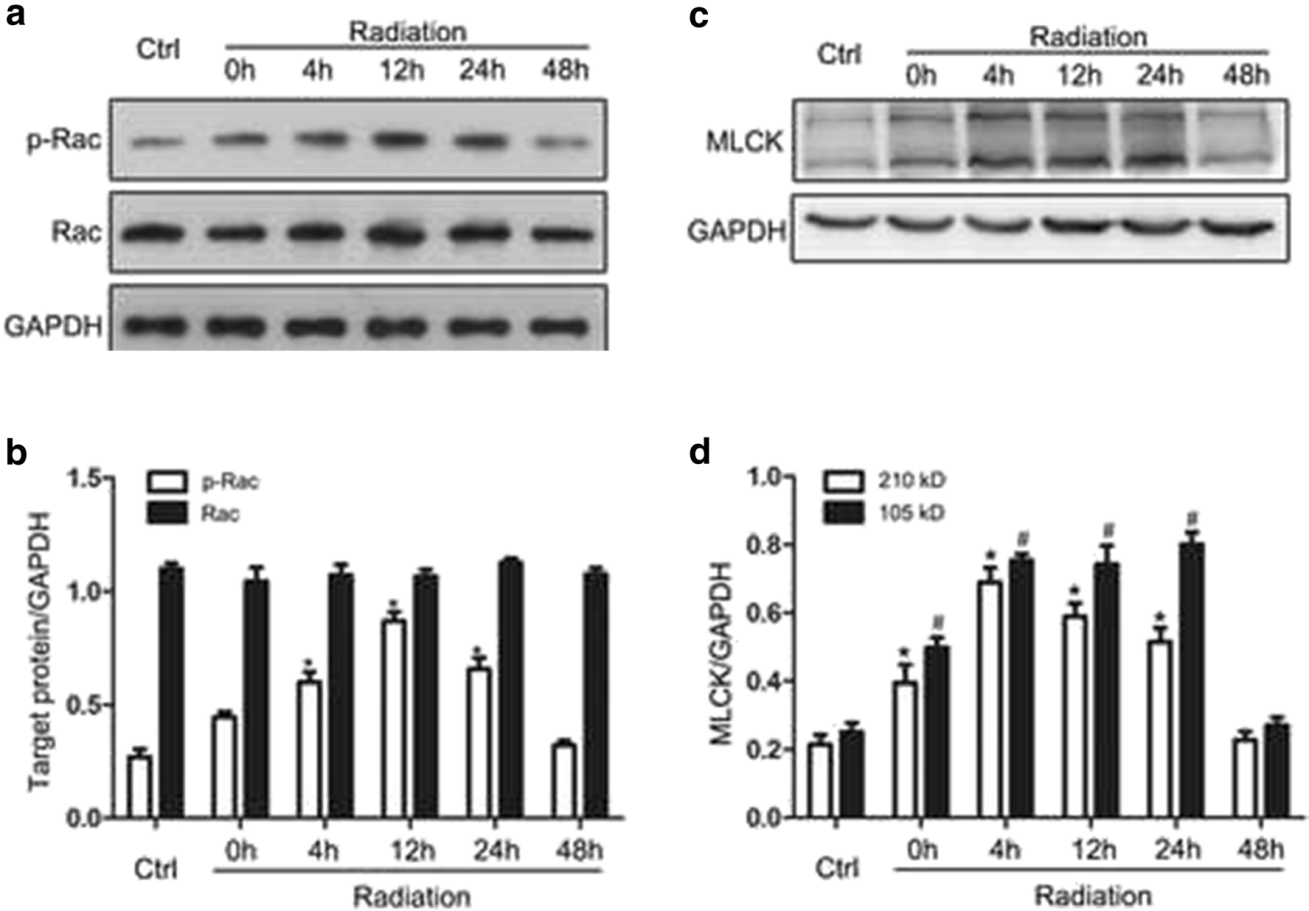
Radiation activates Rac1 and MLCK. **a** Phosphorylated Rac1 (p-Rac1), Rac1 protein expression in primary mouse microglia were determined by Western blotting 0, 4, 12, 24, or 48 h after radiation. **b** Densitometric analyses of p-Rac1 and Rac1 versus GAPDH were shown. Mean ± SEM; *n* = 5; * *P* < 0.05 compared with unirradiated control group. **c** MLCK protein expression in primary mouse microglia were determined by Western blotting 0, 4, 12, 24, or 48 h after radiation. **d** Densitometric analyses of MLCK (210 and 105 kD subunit) versus GAPDH were shown. Mean ± SEM; *n* = 6; * *P* < 0.05, 210 kD subunit of MLCK versus control; # *P* < 0.05, 105 kD subunit of MLCK versus control

**Fig. 7:**
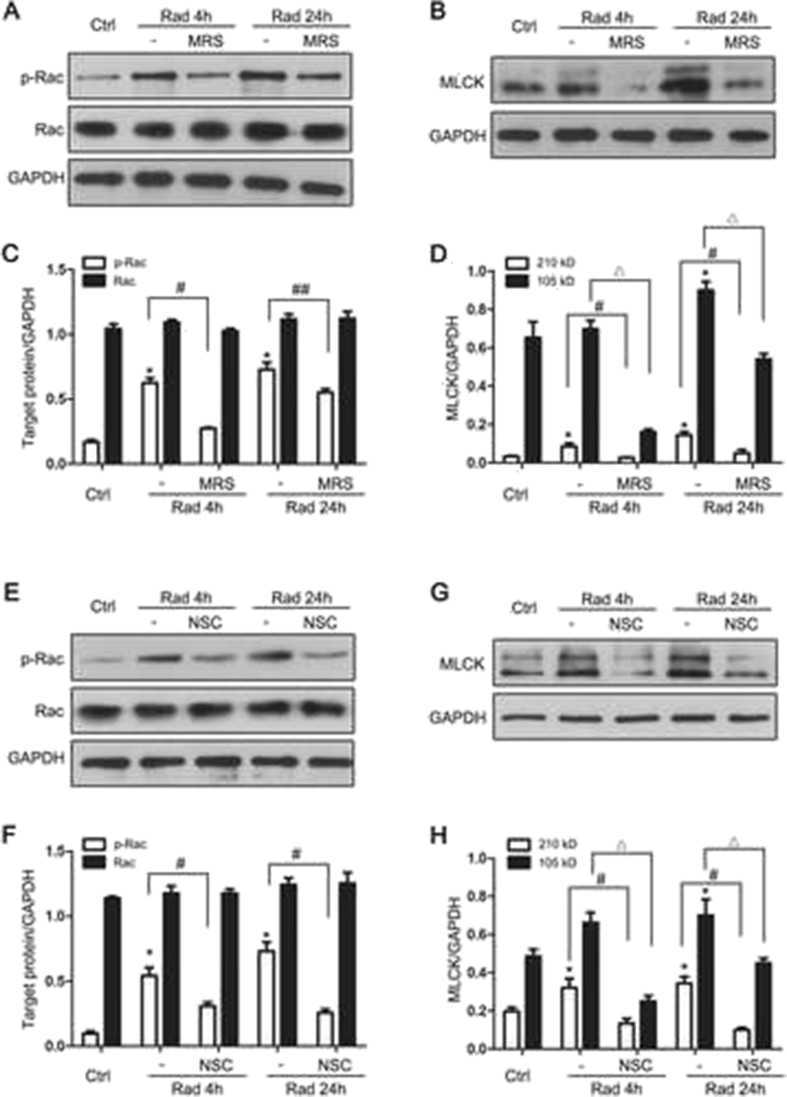
Rac1-MLCK pathway is involved in P2Y6 receptor-mediated microglial phagocytosis after radiation exposure. **a**, **b** p-Rac1, Rac1, and MLCK protein expression in primary mouse microglia were determined by Western blotting 4 h, 24 h after radiation, with or without treatment of MRS2578. **c**, **d** Densitometric analyses of p-Rac1, Rac1, and MLCK versus GAPDH were shown. Mean ± SEM; *n* = 3; * *P* < 0.05 compared with unirradiated control group. # *P* < 0.05 radiated cells pretreated with MRS2578 compared with radiation group 4 h after radiation; ## *P* < 0.01 radiated cells pretreated with MRS2578 compared with radiation group 24 h after radiation. △*P* < 0.05, radiated cells pretreated with MRS2578 compared with radiation group after radiation (105 kD subunit of MLCK); **e**, **g** p-Rac1, Rac1, and MLCK protein expression in primary mouse microglia were determined by Western blotting 4 h, 24 h after radiation, with or without treatment of NSC23766. **f**, **h** Densitometric analyzes of p-Rac1, Rac1, and MLCK versus GAPDH were shown as mean ± SEM; *n* = 3; * *P* < 0.05 compared with unirradiated control group. # *P* < 0.05 radiated cells pretreated with NSC23766 compared with radiation group 4 h after radiation; ## *P* < 0.01 radiated cells pretreated with NSC23766 compared with radiation group 24 h after radiation. △*P* < 0.05, radiated cells pretreated with NSC23766 compared with radiation group after radiation (105 kD subunit of MLCK)

Having demonstrated that the P2Y6 receptor mediates the radiation-induced increase in microglial phagocytosis, which protects neurons by promoting remyelination, we explored the signaling pathways involved in microglial phagocytosis. The initiation of phagocytosis requires the actin cytoskeleton. The actin filaments control the formation of the phagocytic cup, and active Rac1 has been implicated in this signaling cascade [21]. Microglial receptor-mediated phagocytosis is dependent on cytoskeletal MLCK [22]. To determine whether Rac1 and MLCK pathways participate in microglial phagocytosis, we first examined the expression of MLCK and phosphorylated Rac1 in irradiated cultured microglia. As shown in Fig. 6a, b, the level of phosphorylated Rac1 protein expression was increased, peaking 12 h after radiation. By contrast, the expression of total Rac1 did not change. The protein expression of MLCK (two subunits, 210 and 105 kD) also increased after radiation (Fig. 6c, d). These increases in phosphorylated Rac1 (Fig. 7a, c) and MLCK expression (Fig. 7b, d) were both blocked by preincubation with the P2Y6R antagonist MRS2578 (1 μM) in cultured microglia 1 h before radiation exposure, indicating that both Rac1 and MLCK are downstream effectors of the P2Y6R. Treatment with the Rac1 inhibitor NSC23766 [23] markedly decreased the protein expression of phosphorylated Rac1 (Fig. 7e, f) and MLCK (Fig. 7g, h), indicating that MLCK was downstream of Rac1. Taken together, these results indicate that the Rac1-MLCK pathway participates in the radiation-induced P2Y6 receptor-mediated microglial phagocytosis.

## Discussion

**Fig. 8:**
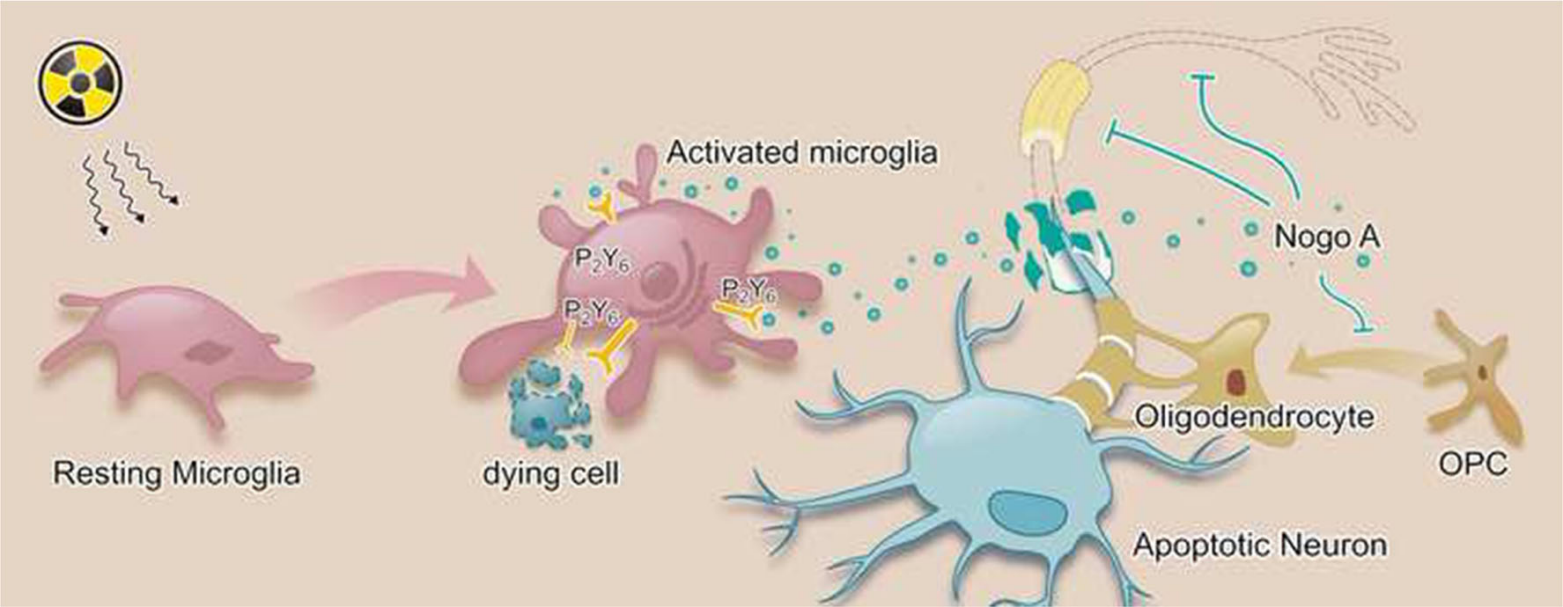
Diagram model for P2X7R mediated microglial activation and phagocytosis in radiation-induced brain injury

Microglial activation plays a critical role in the pathogenesis of radiation-induced brain injury. In this study, we showed that microglial phagocytosis increased after radiation, efficiently removing the inhibitory factor Nogo-A from the myelin debris and engulfing apoptotic neurons. This increase in microglial phagocytosis was paralleled by an increase in the expression of the P2Y6 receptor protein specifically localized to microglia. Inhibition of the P2Y6 receptor with the antagonist MRS2578 suppressed microglial phagocytic capacity, and this was followed by an increase in the number of apoptotic neurons and an exacerbation of the remyelination deficit that occurs after radiation exposure. The clearance of dying cells, myelin debris, and the associated inhibitory factor Nogo-A is important for the recovery of radiation-induced brain injury. We also found that the Rac1-MLCK signaling pathway is involved in the increased P2Y6R-mediated microglial phagocytosis observed in radiation-induced brain injury. The mechanism mediating this increased phagocytosis in brain injury is shown in Fig. 8. First, the efficient removal of apoptotic cells may reduce the release of proinflammatory factors and detrimental substances, such as ATP. Second, the clearance of myelin debris and inhibitory factors such as Nogo-A favors axonal regeneration. In addition, because Nogo-A impedes the differentiation of oligodendrocyte precursor cells to oligodendrocytes, timely removal of Nogo-A may promote remyelination and improve recovery after radiation injury. To our knowledge, this is the first study to investigate the microglial phagocytosis phenotype and its function in radiation-induced brain injury.

Numerous studies have examined the chemotaxis and paracrine effects in microglia; yet, relatively few studies have examined microglial phagocytosis. Thus, whether microglial phagocytosis plays a beneficial or detrimental role in brain diseases remains controversial. Studies have shown that efficient clearance of tissue debris and apoptotic cells is crucial for reconstructing and reorganizing neuronal networking after an insult to the brain [14–16]. In acute brain injury, microglial phagocytosis may benefit axonal regeneration and restoration of the microenvironment, which may help in the recovery from brain injury [24–26]. Our study is consistent with this concept. Yet, Neher and colleagues showed that inhibiting microglial phagocytosis by blocking P2Y6 receptors can prevent neuronal loss and death induced by lipopolysaccharide (LPS) in culture and in vivo, suggesting a detrimental effect of microglial phagocytosis [27]. However, in that study, the pathological models tested were “stressed-but-functional” neurons, which were induced by LPS or lipoteichoic acid, rather than models of neurological disease. Therefore, in their experiment, activated microglia phagocytosed stressed-but-viable neurons, not apoptotic neurons or cell debris. These authors stated that it would also be important to test the function of microglial phagocytosis in animal models of neurological disease. In an animal model of radiation-induced brain injury, there are stressed-but-viable neurons as well as numerous dying cells, myelin debris, inhibitory factors such as Nogo-A, and antigens derived from the dying cells [28–30]. These are the main components phagocytosed by activated microglia. In this scenario, microglia serve as the trash collectors of the brain, helping to remove dying cells and debris to avoid a secondary inflammatory response.

Growing evidence indicates that nucleotides play an important role in neuron-glia communication through P2 purinoceptors, and UDP is recognized as a key molecule in cells associated with many neurodegenerative diseases [31]. In general, phagocytosis requires the activation of several signaling pathways, including the exposure of “eat-me” signals on the target cells and their detection by cell surface receptors on the phagocyte [32]. It was recently demonstrated in animals treated with kainite that UDP released from neurons is required to trigger formation of the phagocytic cup in adjacent microglia, via P2Y6 receptors [33]. However, whether the P2Y6 receptor is involved in radiation-induced brain injury remains unknown, which become the subject of this study. In addition to the P2Y6 receptor, various other receptor types initiate microglial phagocytosis under different treatments, such as toll-like receptors (TLRs) and triggering receptor expressed on myeloid cells 2 (TREM-2). By contributing to phagocytosis, TLRs have been implicated in a variety of cerebral disorders, including bacterial or viral infections, neurodegenerative disorders, inflammatory demyelinating disorders such as multiple sclerosis, and spinal cord injury as well as in development or physiological processes such as neurogenesis, learning, and memory [34–36]. Whether and how TLRs and TREM-2 are involved in radiation-induced brain injury remain to be elucidated in the future.

In epilepsy, Alzheimer’s disease, and Parkinson’s disease, phagocytosis of apoptotic cells is considered beneficial for repairing CNS damage [37]. Myelin proteins, such as Nogo-A, myelin-associated glycoprotein, and oligodendrocyte myelin glycoprotein, translocate to the cell surface at the injured myelin and act as inhibitors of axonal regeneration [38]. Thus, microglial phagocytosis of damaged myelin and cell debris would benefit the survival of injured neurons and myelin regeneration in many neuronal diseases [39, 40]. Indeed, elevated microglial phagocytosis of myelin debris was found to decrease dead cells, resulting in prominent functional recovery [41]. We showed in the present study that when microglial phagocytosis was suppressed, the radiation-induced myelin injury was aggravated, suggesting that phagocytosis promotes neural repair by reducing Nogo-A and by enhancing remyelination. However, a study examining neonatal focal stroke showed that microglial phagocytosis decreases in focal areas with large amounts of neuronal apoptosis [42], plausibly due to the severe damage caused by an overproduction of inflammatory cytokines that inhibit phagocytosis. Therefore, the capacity of microglial phagocytosis varies depending on the stimulation.

Microglial phagocytosis relies on various cell surface receptors and downstream signaling pathways, which ultimately modulate the actin cytoskeleton dynamics and engulfment of damaging debris. A variety of different proteins or signaling pathways that mediate microglial phagocytosis has been reported, such as the TLRs/IRAK4/p38 pathway [43], TREM2/DAP12 pathway [44], or P2Y6 receptor/Ca^2+^ pathway [20]. The signaling pathways that participate in P2Y6R-mediated phagocytosis following radiation-induced brain injury are unclear. Microglial phagocytosis requires the modulation of the cytoskeleton [21]. Previous studies have shown that Rac1 and MLCK participate in the regulation of myosin light chain relaxation and contraction [20]. Rac1, a protein of the Rho family GTPases, regulates both neuronal apoptosis and action cytoskeleton [45]. MLCK is a serine/threonine-specific protein kinase that phosphorylates the regulatory light chain of myosin II. MLCK enables the myosin to cross-bridge with actin filaments and allows contraction to begin. Gitik et al. reported that when MLCK was activated, the nonopsonized myelin was phagocytosed effectively [22]. We showed here that Rac1 and MLCK were involved in P2Y6R-mediated microglial phagocytosis in radiation-induced brain injury.

In summary, our results provided the first evidence of microglial phagocytosis in radiation-induced brain injury in vitro and in vivo. We showed that P2Y6 receptor-mediated phagocytosis benefits damaged neurons by the efficient removal of apoptotic cells, engulfment of myelin debris, and promotion of remyelination. The Rac1/MLCK pathway lies downstream of the P2Y6 receptor in these processes. We concluded that P2Y6 receptor-mediated microglial phagocytosis in radiation-induced brain injury provides a new insight for therapeutic intervention to restore neuronal survival and brain function.

## References

[1] Welsh LC, Dunlop AW, McGovern T, McQuaid D, Dean JA, Gulliford SL, Bhide SA, Harrington KJ (2014). Neurocognitive function after (chemo)-radiotherapy for head and neck cancer. Clin Oncol.

[2] Ampil FL, Kim DD, Ghali GE, Baluna RG (2012). How intensive should radiotherapy for head and neck cancer with synchronous distant metastases be? Review of cases. J Oral Maxillofacl Surg: Off J Am Assoc Oral Maxillofac Surg.

[3] Butler JM, Rapp SR, Shaw EG (2006). Managing the cognitive effects of brain tumor radiation therapy. Curr Treat Options in Oncol.

[4] McIver JI, Pollock BE (2004). Radiation-induced tumor after stereotactic radiosurgery and whole brain radiotherapy: case report and literature review. J Neuro-Oncol.

[5] Dietrich J, Monje M, Wefel J, Meyers C (2008). Clinical patterns and biological correlates of cognitive dysfunction associated with cancer therapy. Oncologist.

[6] Wang YX, King AD, Zhou H, Leung SF, Abrigo J, Chan YL, Hu CW, Yeung DK (2010). Evolution of radiation-induced brain injury: MR imaging-based study. Radiology.

[7] Greene-Schloesser D, Robbins ME, Peiffer AM, Shaw EG, Wheeler KT, Chan MD (2012). Radiation-induced brain injury: a review. Front Oncol.

[8] Shaw EG, Robbins ME (2006). The management of radiation-induced brain injury. Cancer Treat Res.

[9] Liu B, Hong JS (2003). Role of microglia in inflammation-mediated neurodegenerative diseases: mechanisms and strategies for therapeutic intervention. J Pharmacol Exp Ther.

[10] Gonzalez-Scarano F, Baltuch G (1999). Microglia as mediators of inflammatory and degenerative diseases. Annu Rev Neurosci.

[11] Choi DK, Koppula S, Suk K (2011). Inhibitors of microglial neurotoxicity: focus on natural products. Molecules.

[12] Fu R, Shen Q, Xu P, Luo JJ, Tang Y (2014). Phagocytosis of microglia in the central nervous system diseases. Mol Neurobiol.

[13] Kotter MR, Li WW, Zhao C, Franklin RJ (2006). Myelin impairs CNS remyelination by inhibiting oligodendrocyte precursor cell differentiation. J Neurosci: Off J Soc Neurosci.

[14] Brown GC, Neher JJ (2014). Microglial phagocytosis of live neurons. Nat Rev Neurosci.

[15] Giunta B, Ehrhart J, Obregon DF, Lam L, Le L, Jin J, Fernandez F, Tan J (2011). Antiretroviral medications disrupt microglial phagocytosis of beta-amyloid and increase its production by neurons: implications for HIV-associated neurocognitive disorders. Mol Brain.

[16] Iadecola C, Anrather J (2011). The immunology of stroke: from mechanisms to translation. Nat Med.

[17] Peng Y, Lu K, Li Z, Zhao Y, Wang Y, Hu B, Xu P, Shi X (2014). Blockade of Kv1.3 channels ameliorates radiation-induced brain injury. Neuro-Oncology.

[18] Panagiotakos G, Alshamy G, Chan B, Abrams R, Greenberg E, Saxena A, Bradbury M, Edgar M (2007). Long-term impact of radiation on the stem cell and oligodendrocyte precursors in the brain. PLoS One.

[19] Olschowka JA, Kyrkanides S, Harvey BK, O’Banion MK, Williams JP, Rubin P, Hansen JT (1997). ICAM-1 induction in the mouse CNS following irradiation. Brain Behav Immun.

[20] Koizumi S, Shigemoto-Mogami Y, Nasu-Tada K, Shinozaki Y, Ohsawa K, Tsuda M, Joshi BV, Jacobson KA (2007). UDP acting at P2Y6 receptors is a mediator of microglial phagocytosis. Nature.

[21] Niedergang F, Chavrier P (2005). Regulation of phagocytosis by Rho GTPases. Curr Top Microbiol Immunol.

[22] Gitik M, Reichert F, Rotshenker S (2010). Cytoskeleton plays a dual role of activation and inhibition in myelin and zymosan phagocytosis by microglia. FASEB J: Off Publ Fed Am Soc Exp Biol.

[23] Gao Y, Dickerson JB, Guo F, Zheng J, Zheng Y (2004). Rational design and characterization of a Rac GTPase-specific small molecule inhibitor. Proc Natl Acad Sci U S A.

[24] Dubois-Dalcq M, Ffrench-Constant C, Franklin RJ (2005). Enhancing central nervous system remyelination in multiple sclerosis. Neuron.

[25] Franklin RJ, Kotter MR (2008). The biology of CNS remyelination: the key to therapeutic advances. J Neurol.

[26] Fadok VA, Bratton DL, Konowal A, Freed PW, Westcott JY, Henson PM (1998). Macrophages that have ingested apoptotic cells in vitro inhibit proinflammatory cytokine production through autocrine/paracrine mechanisms involving TGF-beta, PGE2, and PAF. J Clin Invest.

[27] Neher JJ, Neniskyte U, Hornik T, Brown GC (2014). Inhibition of UDP/P2Y6 purinergic signaling prevents phagocytosis of viable neurons by activated microglia in vitro and in vivo. Glia.

[28] Block ML, Hong JS (2005). Microglia and inflammation-mediated neurodegeneration: multiple triggers with a common mechanism. Prog Neurobiol.

[29] Rock RB, Peterson PK (2006). Microglia as a pharmacological target in infectious and inflammatory diseases of the brain. J Neuroimmune Pharmacol: Off J Soc NeuroImmune Pharmacol.

[30] Greter M, Merad M (2013). Regulation of microglia development and homeostasis. Glia.

[31] Ekdahl CT, Kokaia Z, Lindvall O (2009). Brain inflammation and adult neurogenesis: the dual role of microglia. Neuroscience.

[32] Ravichandran KS (2011). Beginnings of a good apoptotic meal: the find-me and eat-me signaling pathways. Immunity.

[33] Inoue K (2007). UDP facilitates microglial phagocytosis through P2Y6 receptors. Cell Adhes Migr.

[34] Marsh BJ, Williams-Karnesky RL, Stenzel-Poore MP (2009). Toll-like receptor signaling in endogenous neuroprotection and stroke. Neuroscience.

[35] Hanke ML, Kielian T (2011). Toll-like receptors in health and disease in the brain: mechanisms and therapeutic potential. Clin Sci.

[36] Ziegler G, Harhausen D, Schepers C, Hoffmann O, Rohr C, Prinz V, Konig J, Lehrach H (2007). TLR2 has a detrimental role in mouse transient focal cerebral ischemia. Biochem Biophys Res Commun.

[37] Sierra A, Abiega O, Shahraz A, Neumann H (2013). Janus-faced microglia: beneficial and detrimental consequences of microglial phagocytosis. Front Cell Neurosci.

[38] Yiu G, He Z (2006). Glial inhibition of CNS axon regeneration. Nat Rev Neurosci.

[39] Boekhoff TM, Ensinger EM, Carlson R, Bock P, Baumgartner W, Rohn K, Tipold A, Stein VM (2012). Microglial contribution to secondary injury evaluated in a large animal model of human spinal cord trauma. J Neurotrauma.

[40] Cullheim S, Thams S (2007). The microglial networks of the brain and their role in neuronal network plasticity after lesion. Brain Res Rev.

[41] Bose S, Cho J (2013). Role of chemokine CCL2 and its receptor CCR2 in neurodegenerative diseases. Arch Pharm Res.

[42] Faustino JV, Wang X, Johnson CE, Klibanov A, Derugin N, Wendland MF, Vexler ZS (2011). Microglial cells contribute to endogenous brain defenses after acute neonatal focal stroke. J Neurosci: Off J Soc Neurosci.

[43] Lee CY, Landreth GE (2010). The role of microglia in amyloid clearance from the AD brain. J Neural Transm.

[44] Satoh J, Tabunoki H, Ishida T, Yagishita S, Jinnai K, Futamura N, Kobayashi M, Toyoshima I (2012). Phosphorylated Syk expression is enhanced in Nasu-Hakola disease brains. Neuropathol: Off J Jpn Soc Neuropathol.

[45] Kataoka A, Koga Y, Uesugi A, Tozaki-Saitoh H, Tsuda M, Inoue K (2011). Involvement of vasodilator-stimulated phosphoprotein in UDP-induced microglial actin aggregation via PKC- and Rho-dependent pathways. Purinergic Signal.

